# Potential consequences of climate change for primary production and fish production in large marine ecosystems

**DOI:** 10.1098/rstb.2012.0231

**Published:** 2012-11-05

**Authors:** Julia L. Blanchard, Simon Jennings, Robert Holmes, James Harle, Gorka Merino, J. Icarus Allen, Jason Holt, Nicholas K. Dulvy, Manuel Barange

**Affiliations:** 1 Department of Animal and Plant Sciences, University of Sheffield, Western Bank, Sheffield S10 2TN, UK; 2 Division of Biology, Imperial College London, Silwood Park Campus, Buckhurst Road, Ascot SL5 7PY, UK; 3 Centre for Environment Fisheries and Aquaculture Science, Pakefield Road, Lowestoft NR33 0HT, UK; 4 School of Environmental Sciences, University of East Anglia, Norwich NR4 7TJ, UK; 5 Plymouth Marine Laboratory, Prospect Place, Plymouth PL1 3DH, UK; 6 National Oceanography Centre – Liverpool, 6 Brownlow Street, Liverpool L3 5DA, UK; 7 Earth to Ocean Research Group, Department of Biological Sciences, Simon Fraser University, 8888 University Drive, Burnaby, British Columbia, CanadaV5A 1S6

**Keywords:** global environmental change, benthic–pelagic coupling, fisheries ecology, marine macroecology, marine communities, size spectrum

## Abstract

Existing methods to predict the effects of climate change on the biomass and production of marine communities are predicated on modelling the interactions and dynamics of individual species, a very challenging approach when interactions and distributions are changing and little is known about the ecological mechanisms driving the responses of many species. An informative parallel approach is to develop size-based methods. These capture the properties of food webs that describe energy flux and production at a particular size, independent of species' ecology. We couple a physical–biogeochemical model with a dynamic, size-based food web model to predict the future effects of climate change on fish biomass and production in 11 large regional shelf seas, with and without fishing effects. Changes in potential fish production are shown to most strongly mirror changes in phytoplankton production. We project declines of 30–60% in potential fish production across some important areas of tropical shelf and upwelling seas, most notably in the eastern Indo-Pacific, the northern Humboldt and the North Canary Current. Conversely, in some areas of the high latitude shelf seas, the production of pelagic predators was projected to increase by 28–89%.

## 1. Introduction

Globally, some 80 Mt of marine fish are landed each year and these are consumed directly or used to produce fishmeal and oils for aquaculture and agriculture [1]. Many people are reliant on fish as their primary protein source in poorer countries, increasing their vulnerability to changes in fisheries production [2]. Burgeoning coastal human populations are also expected to increase demands for fish in future, and the potential production of fisheries will be influenced by climate change [3–5]. This poses challenges for the management of marine ecosystems and fisheries, especially when fish populations are already overexploited and/or climate reduces production [1,6].

Climate change influences fisheries production through its effects on primary production, food web interactions and the life history and distribution of target species. Changes in primary production follow from changes in the physical and chemical environment [7], while changes in the food web are also influenced by the availability of primary production [8]. Empirical evidence for climate change effects on marine ecosystems and their component species is growing [9–11]. Also, over the past three decades, the waters of the northeast Atlantic have warmed faster than the global average, and the distributions [12,13] and relative abundance of fishes have changed on local and regional scales [14]. Because the many impacts of climate change on marine ecosystems may be additive, synergistic or antagonistic, it is challenging to understand and predict responses at all spatial scales and levels of ecological complexity [15–17].

Climate effects on total fisheries production are particularly challenging to predict at ecosystem scales. Species identities, habitat affinities, physiological preferences, life histories and interspecific interactions vary among systems and are rarely known in sufficient detail to make predictions. As a result of this, we have tended to see the emergence of predictions for a few species at ecosystem scales, especially where the physical environment, the species' ecology and interactions with the environment were previously well studied [18,19], and predictions based on simpler principles, such as climate envelope approaches, that can be applied at global scales [20,21].

Our limited understanding of the ecology of all species that contribute to fisheries production impedes our capacity to use species-based models to predict changes in fisheries production with climate change. As well as further developing species-focused approaches [22], an alternative and potentially informative parallel approach is to develop methods that do not require knowledge of individual species' ecology. One promising method is based on size-based analyses that can be used to assess the consequences of changes in the environment and primary production changes on abundance and production at higher trophic levels [23]. The strengths of size-based analyses are that marine food webs are structured by size as much as species identity, because most primary producers are small, and that the body sizes of consumers are linked to their position in the food web. Further, body size largely determines metabolic rate and hence the rates of individual consumption and production [24,25].

Static size-based models that are based on metabolic theory and empirical relationships between body size and trophic level have been applied to investigate unexploited production and biomass of larger marine animals in the global oceans under current environmental conditions [26]. Dynamic size-spectrum models can extend this approach by considering the time-dependent and continuous growth and mortality processes that result from size-structured feeding, representative of pelagic ecosystems [27,28]. They can be used to predict the consequences of fishing mortality and changes in primary production as well as temperature effects on dynamical changes in the community size spectrum [29,30], and have been modified to incorporate the strong benthic–pelagic coupling that characterizes relatively shallow aquatic ecosystems, including shelf seas [31–33]. Despite simple underlying assumptions and relative ease of parametrization, size-based models have proved remarkably capable of predicting broad-scale patterns of size-structure and production observed in nature [26,31].

Most of the global fish catch is taken from countries' exclusive economic zones (EEZ) in shelf-sea ecosystems, where most of the global marine primary production also occurs. Thus, any effects of climate on these seas are expected to have disproportionate effects on global primary and fish production. However, the current generation of coupled global climate models are unable to resolve many of the processes that influence the primary production in shelf sea ecosystems (e.g. tides, wind, run-off, recycling, seasonal stratification). To begin to address some of these issues, high-resolution-coupled physical–biogeochemical models of the shelf seas are being used, where downscaled data from the global climate simulations are used to force these regional models to project future changes in shelf-sea ecosystems [34].

One such high-resolution-coupled physical–biogeochemical model has been extensively evaluated for parts of the Northwest European Shelf and has recently been extended for application to the global coastal ocean [34–38]. It is composed of two components: (i) the Proudman Oceanographic Laboratory Coastal-Ocean Modelling System—a three-dimensional physical hydrodynamic model, and (ii) the European Seas Regional Ecosystem Model, which describes the biogeochemical processes affecting the flow of carbon, nitrogen, phosphorus and silicon in planktonic pelagic and benthic ecosystems. The major controls on phytoplankton primary production in the model are the availability of light and nutrients and grazing pressure. On seasonal time-scales, the interplay between the light climate, turbulence and density stratification influences phytoplankton growth. On annual to multi-decadal time-scales, oceanic, terrestrial and atmospheric coupling control the nutrient supply to the shelf seas and hence the phytoplankton growth.

Here, we combine physical–biogeochemical and size-based ecosystem models to project future effects of climate change on fish biomass and production in 11 large regional domains. These include many of the most productive areas of the shelf seas and encompass 28 large marine ecosystems and 107 EEZ and adjacent areas. This area currently yields 60 per cent of average annual global catch and 77 per cent of total catch from inside EEZ. We use global climate model data and the ‘business as usual’ scenario from the Intergovernmental Panel on Climate Change (IPCC) Special Report on Emissions Scenarios (SRESA1B) [39] to force the whole biophysical modelling system, from which we obtain size-based estimates of changes in production and biomass by 2050. Projected ecosystem states in the absence of fishing are compared with those using a static size-based model of energy flux from primary producers to consumers. To validate fisheries past catch projections, recorded catches are compared with modelled catches for 78 of the countries EEZ. Finally, we explore the combined consequences of different fishing and climate change scenarios, to determine their relative effects within and among ecosystems.

## 2. Methods

### (a) Physical–biogeochemical model and climate change scenarios

The coupled physical–biogeochemical models were set up and run for 11 large regional domains. All models had a horizontal resolution of 1/10° horizontal and 42 vertical layers. The high-resolution results were aggregated by EEZ (107 areas within the 11 domains). The EEZ was deemed to be the most relevant spatial unit for predicting change in fisheries. Within EEZ, we modelled the dynamics of consumers using a size-structured food web approach (described below).

Two climate *in silico* experiments were carried out for each of the 11 domains, forced with ocean and atmosphere data taken from the Institut Pierre Simon Laplace Climate Model run for the Fourth Assessment Report of the IPCC [39]. The first, a present-day control experiment, used data from a simulation forced with trace gases set to 1980 values. The second, a near-future climate experiment (*ca* 2050) was performed using data taken from the IPCC SRESA1B ‘business as usual’ emissions simulation. Using forcing data for two different time periods from the same climate model enables relative changes between the two experiments to be quantified. In addition, a re-analysis simulation was performed for the period 1992–2001. Forcing data were provided by a global ocean assimilation and re-analysis simulation [40] and an atmospheric re-analysis dataset (http://badc.nerc.ac.uk/data/ecmwf-e40/). This enabled the outputs from the coupled hydrodynamic-ecosystem model to be evaluated against oceanographic and fisheries data for the same period. Nutrient input from riverine sources was provided by the Global Nutrient Export from Water Sheds model [41]. These data were used for both sets of climate model forced experiments and also for the re-analysis experiments, thus removing any climate change signal from riverine inputs. This was carried out because there were no reliable projections for riverine nutrient inputs available for the 11 domains. For each experiment, we ran a total of 13 years of simulations, with the final 10 used to capture both the signal and variability.

### (b) Size-structured community models with temperature effects

The size-structured dynamics of marine animal communities were modelled using a previously published size-based model [31,32], which was modified to incorporate a temperature effect on the feeding and intrinsic mortality rates of organisms. The model incorporates two coupled size-structured communities that have distinct trophic properties: ‘pelagic predators’ and ‘benthic detritivores’. In both communities, we are concerned with the continuous function *N*(*m*,*t*) (m^−3^ g^−1^) which gives the density per unit mass per unit volume for organisms of mass *m* at time *t*. The continuous processes of growth *G* and mortality *D* that arise from organisms encountering and eating available and suitable food govern the temporal dynamics and lead to a partial differential equation for each size spectrum *i*, where *i* is denoted as P = pelagic predators or B = benthic detritivores (see electronic supplementary material, tables S1 and S2 for equations and parameters).

In the pelagic community, there is a background ‘plankton’ spectrum that spans the size range from 10^−12^ to 10^−3^ g that forms part of the food for ‘pelagic predators’. The feeding rate *f_Pi_*(*m*,*t*) of a given size pelagic predator is a function of the preference for prey **ω*_i_* in spectrum *i*, the volume of water searched per unit time 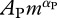 (m^3^ yr^−1^ ; where *A* is the relative volume of water searched rate per unit body mass *m* and **α** is the allometric scaling exponent), and the amount of suitably sized food available in spectrum *i*. The probability of a predator of size *m* eating an encountered prey of size *m*′ is given by a lognormal probability density function, with a mean value representing the preferred predator–prey mass ratio and a standard deviation that represents the breadth of the relative prey mass. Realized predator–prey mass ratios in fish communities do not vary systematically with temperature or primary production in the world's oceans [42]. Benthic consumers compete for an unstructured shared pool of food and do not feed according to prey size. For simplicity, we call the latter group ‘detritivores’ because in most benthic invertebrate communities detritus forms the bulk of their food (but it could also be supplemented by living phytoplankton). The feeding rate of a detritivore *f*_B_(*m*,*t*) depends on the volume of water either searched or filtered per unit time 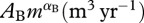 and the available biomass density of detritus *B*_D_(*t*) (g m^−3^).

A temperature effect on feeding and intrinsic mortality rates was incorporated into the model to enable the effects of changes in temperature to be assessed. The temperature effect was based on the Arrhenius function, **τ** = e*^c^*^1 −^ *^E^*^/(*kT*)^, where *c*1 is a constant (25.55), *E* is the activation energy of heterotrophic metabolism (0.63 eV), *T* is temperature in kelvin (°C + 273) and *k* is the Boltzmann constant (8.62×10^−5^ eV K^−1^) [43]. Feeding rates drive the dynamic processes of growth and predation mortality. Other size-dependent (but temporally constant) sources of mortality include intrinsic natural mortality, senescence and fishing mortality.

The size-based model was forced with outputs (daily phytoplankton, microzooplankton and detritus biomass density, sea surface and sea floor temperature) from the physical–biogeochemical model. The size-based model was applied to all EEZ using the same parameter values, such that only the forcing variables differed among EEZ. For each EEZ and scenario, the model was first run to equilibrium using time-averaged input before applying the model to time-varying environmental conditions for the duration of a 10-year time slice, under each of the scenarios. We labelled and computed the numerical density, biomass density, production and catch across the pelagic predator size range from 1.25 g to 100 kg as ‘fish’, because fish typically dominate biomass in this size range (see the electronic supplementary material for further details).

To predict changes in fishing effects and catches, a series of fishing scenarios were run for all modelled EEZ, across all time slices and climate scenarios. An ‘even’ fishing selectivity scenario was applied uniformly across size classes, such that all organisms greater than 1.25 g had the same fishing mortality (*F*). This was intended to represent fisheries targeting species for fishmeal production (small pelagic, 1.25–80 g) and direct human consumption (larger pelagic and demersal, 80 g–100 kg).

Although at steady state, the modelled size spectra follow a power law scaling with numerical density and body mass, the effects of fishing and other drivers can sometimes result in nonlinearities. To measure the disruption of the size spectra from fishing and climate change effects, the total deviation between the impacted size spectrum log*N*_I_(*m*) and the unexploited steady state size spectrum log*N*_U_(*m*) across the fish size range was measured according to Law *et al*. [44]:2.18
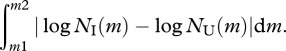


### (c) Model validation and data

To validate the predictions of our coupled model against data, we forced the size-based model for the period 1992–2001 with Ocean and Atmospheric reanalysis datasets used to provide boundary conditions in the physical–biogeochemical model. Fish production estimates at *F* = 0.8 yr^−1^ were compared with national catch statistics from the United Nations Food and Agriculture Organisation (FAO) database for 78 EEZ that overlapped with those captured in the 11 oceanic domains used in our analysis (see the electronic supplementary material, table S3).

For estimating current total catches, we assumed a size-specific fishing mortality rate *F* = 0.8 yr^−1^. This value was intended to represent high sustainable rates of fishing on smaller species that dominate total catches for the EEZ. For exploring the relative effects of climate and fishing scenarios, we also used a lower value *F* = 0.2 yr^−1^ expected to be more sustainable at the community level.

For modelled communities in the absence of fishing, the results from the dynamic model were compared with those from a static size-based scaling model of energy flux from primary producers to consumers [26]. Jennings *et al*.'s [26] static scaling model uses principles from macroecology, life-history theory and food web ecology to predict the potential biomass, production, size and trophic structure of consumer communities. In the static scaling model, temperature is assumed to act on metabolic rate and hence individual production. Both the static and dynamic models were forced with the same predictions for the physical environment and we assumed the same temperature-independence of the predator–prey mass ratio.

## 3. Results

### (a) Predicted effects of climate change on unexploited marine ecosystems

**Figure 1.**
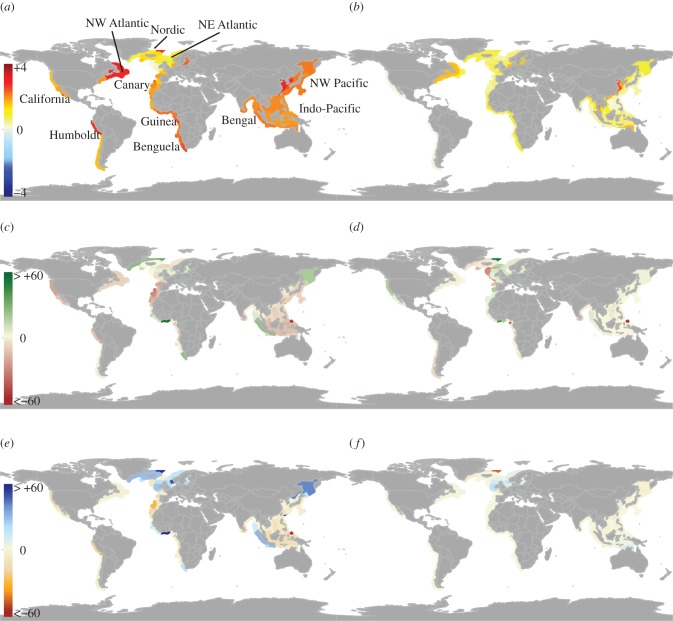
Mean-predicted relative changes for 2050 under the SREASA1B scenario. Maps of change in (*a*) mixed-layer depth temperature; (*b*) near sea floor temperature (°C); and percentage changes in: (*c*) density of phytoplankton and (*d*) biomass density of detritus; (*e*) biomass density of pelagic predators and (*f*) biomass density of benthic detritivores.

The predicted bottom-up effects of climate change, in the absence of fisheries exploitation, varied widely among EEZ (figure 1). In general, changes in fish production and biomass density mirrored the changes in primary production and phytoplankton density more strongly than changes in temperature. Although the greatest warming was predicted in the EEZ of China, South Korea and along the east coast of North America, only modest reductions in primary production and phytoplankton density (−6%), occurred in these areas, resulting in small changes in overall biomass density of fish (−6%). The largest predicted reductions in phytoplankton and zooplankton density occurred in EEZ within the Indo-Pacific (Palau, −60%), the Humboldt Current (Peru, Chile/Peru, −35%) and the Canary Current (Madeira) regions and caused similar magnitudes of change in the overall biomass of fish. At the other extreme, the largest increases in phytoplankton and zooplankton biomass density led to the largest increases in fish biomass density. Such was the case for EEZ of the Guinea Current (Ghana, Ivory coast, Togo) and the Nordic shelf seas (Jan-Mayen, Greenland), where overall fish biomass density increases exceeded 30 per cent. In some cases, the predicted effects of changes in phytoplankton biomass density on fish density were countered or enhanced by changes in the detritus and benthic detritivore pathway, in zooplankton, or by a very large increase in temperature.

In the absence of exploitation, the predictions of the dynamic size spectra model were closely correlated with those from the static size-based model (Spearman's rank correlation, **ρ** = 0.88; electronic supplementary material, figure S1). Outliers occurred because the more simplistic static model did not account for a benthic detritus energy pathway that was predicted to account for a significant proportion of energy flux in some ecosystems.

### (b) Top-down effects: validation of the model with fishing

**Figure 2.**
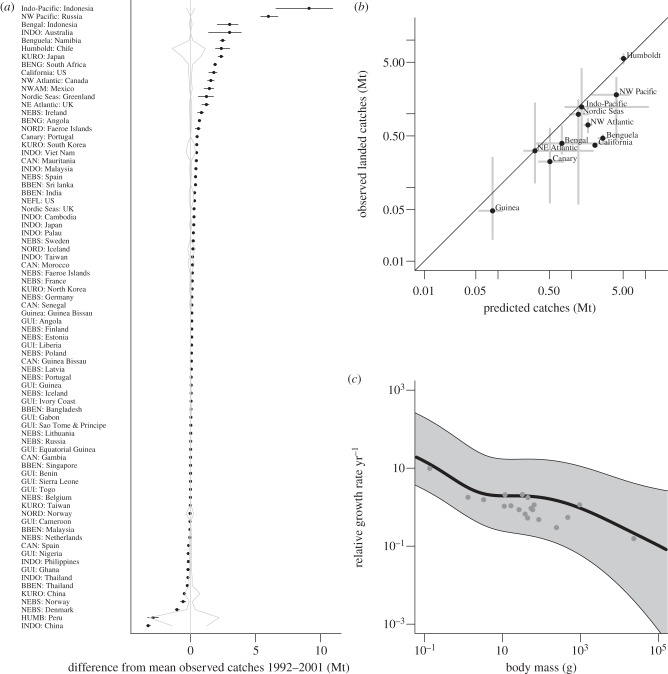
Comparison of model results with data. (*a*) Differences from mean observed values of fisheries landings averaged over the 1992–2001 from 11 large regional domains, grouped by 78 country EEZ. Grey lines indicate range of interannual variation in the observed fisheries landings over 1992–2001 data, whereas error bars show the range of interannual values predicted by the model. (*b*) Relationship between modelled catches (Mt per year) and the observed landed catches aggregated to the domain level. The points show the median across all EEZ within each domain and the grey lines show the extent from the 25th to the 75th percentiles. Solid line is 1 : 1 relationship. (*c*) Mean modelled relative growth rates over 1992–2001 across all EEZ (grey areas, mean for northeast Atlantic shown in central line) along with relative growth rates at 10% of asymptotic size estimated from empirical von Bertalanffy growth equations for a subset of the fish populations from the northeast Atlantic given in Pauly [45].

When fishing mortality was added to the dynamic model for the period 1992–2001, modelled catches from 78 of the 107 EEZ were comparable to reported catches (figure 2*a*). Because true rates of community-wide fishing mortality and selectivity were not known, we assumed a fishing mortality rate of 0.8 yr^−1^ for all fished size classes, consistent with fishers heavily exploiting all fish that were present. The greatest discrepancies between predicted and reported catches were those for EEZ within the Indo-Pacific (Indonesia) and Northwest Pacific shelf sea (the Sea of Okhotsk, off Russia) regions, where predicted fisheries catches were more than 5 Mt greater than the mean reported catches. The largest deviations from reported catches were also associated with high interannual variability in both model- and data-based catch estimates, for example, for Peru and Chile EEZ. When catches and predictions were aggregated at the domain level across EEZ, the 50 per cent quantiles of predicted catches were strongly correlated with the 50 per cent quantiles of reported catches (figure 2*b*; Spearman's rank was **ρ** = 0.8 for median catches at the domain level, compared with **ρ** = 0.63 for mean catches averaged over 1992–2001 at the EEZ level).

Modelled relative growth rates were also realistic and fell within the range of empirical growth rates of fish species from the North Sea (figure 2*c*). The highest relative growth rates occurred in warm and highly production regions (Bay of Bengal), whereas the slowest relative growth rates occurred in cold and lower production ecosystems (Nordic Shelf Seas), in line with expectations from food availability and temperature effects on growth.

### (c) Top-down and bottom-up: relative effects of fishing and climate change

**Figure 3.**
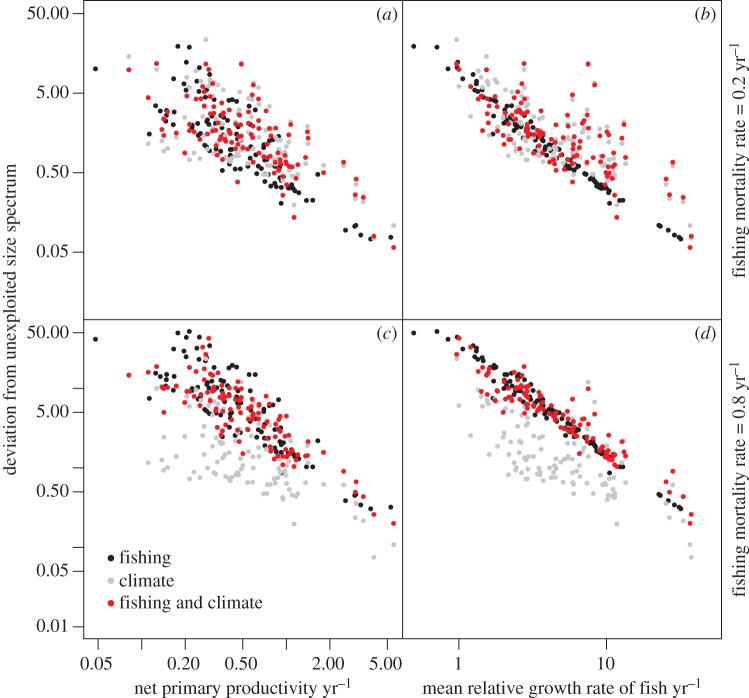
Across-ecosystem effects of fishing and climate change. Deviation from the unexploited size spectrum versus mean net primary productivity (*a*,*c*) and mean relative growth rates of fish (*b*,*d*) when ecosystems are subjected to: fishing (black), climate change (grey) and climate and fishing (red). Each point represents an EEZ. Equilibrium results based on time-averaged environmental conditions under each scenario were used. Results shown for (*a*,*b*) low (0.2 yr^−1^) and (*c*,*d*) high fishing mortality rates (0.8 yr^−1^). Note the logarithmic scale markings on all of the axes.

Ecosystems responded differently to the same fishing scenarios in the absence of climate change. At the EEZ level, the disruption to the size spectrum from fishing impacts was smaller when net primary production and mean relative growth rates of fish were higher (figure 3). There was a stronger relationship with the latter because realized fish growth rates integrated the effects of both temperature and food availability. Without fishing and only climate change, there was greater variation in the relationship between the disruption to the size spectrum and primary production owing to the mixed responses and multiple environmental drivers under the climate change scenario. The combined effects of both fishing and climate drivers depended on how heavily fished the community was. Under the low fishing mortality rate of 0.2 yr^−1^, climate effects dominated the deviation from the unexploited size spectrum, whereas fishing effects dominated when mortality rates were high, 0.8 yr^−1^.

**Figure 4.**
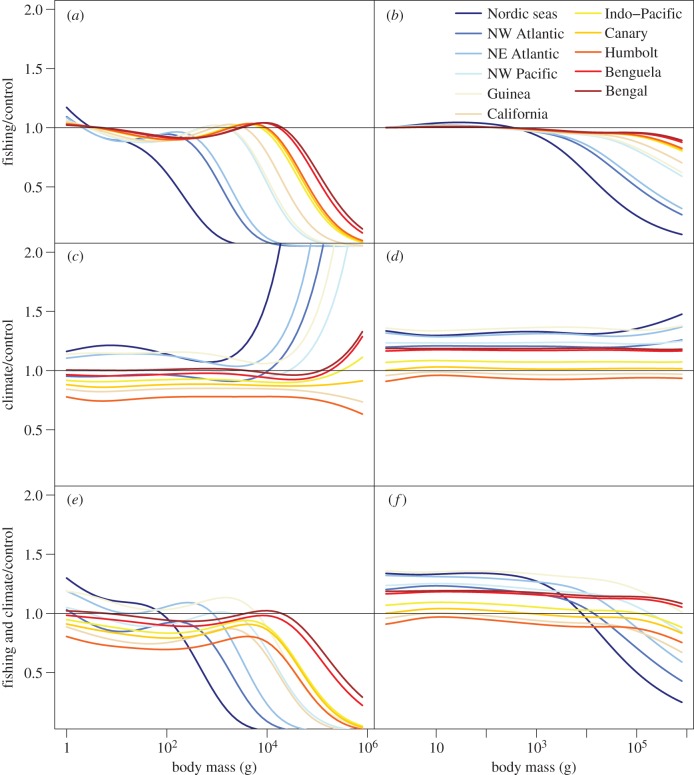
Changes in community size structure from fishing and climate. Changes in density at size (*a*,*c*,*e*) and relative growth rates at size (*b*,*d*,*f*) relative to unexploited control size spectra for (*a*,*b*) fishing, (*c*,*d*) climate and fishing and (*e*,*f*) climate effects. Results shown are for heavy fishing mortality rates only (0.8 yr^−1^). Size spectra were averaged across each of the 11 large regional domains. The domains are ranked in the legend according to fish growth rates (from lowest in the Nordic seas to highest in Bay of Bengal).

Under heavy fishing pressure, reductions in the numerical density relative to the unexploited steady state were most pronounced at larger body sizes (figure 4) and in the domains where individual growth rates were slowest (e.g. Nordic seas, northwest Atlantic and northeast Atlantic shelf seas). Climate change increased or decreased numerical density and growth rates relative to the unexploited steady state across all sizes. The greatest increases occurred in the Nordic seas, northwest Atlantic, northeast Atlantic shelf seas and Gulf of Guinea and the greatest decreases were in the Humboldt, Canary and California current ecosystems. If increases in primary production occurred, the combined effects of climate and fishing relative to those in an unexploited ecosystem were less than fishing alone, but also resulted in stronger top-down cascading effects along the size spectrum. If reductions in primary production occurred, the response of fishing with climate change was magnified (figure 4).

The effect of heavy fisheries exploitation on the resilience of ecosystems to climate change was examined by comparing the coefficients of variation from the biomass density time-series for the most sensitive ‘large’ fish component of the size spectrum (e.g. across the 80 g–100 kg size range), generated from seasonal and interannual variability within each of the EEZ, scenario time slices and fishing mortalities. Overall, the variability in biomass density increased with higher fishing mortality and when the combined effects of fishing and climate reduced fish production (see the electronic supplementary material, figure S2).

## 4. Discussion

This study is a first, and necessarily simplified, attempt towards understanding the potential consequences of climate change on large marine ecosystems and their fisheries using a dynamical size-based food web approach. We achieve this by linking the trophic ecology of coupled size-structured communities with predicted changes in the physical and biogeochemical environment. The dynamic size-spectrum model predicts a mixture of positive and negative responses in fish biomass density and production that mirror the predicted changes in primary production more strongly than changes in temperature. The results corroborate empirical work by showing that potential marine fisheries production is primarily governed by available primary production [46–48].

Previous studies have predicted 30–70% average increases in potential fish production at high latitudes and decreases of up to 40 per cent in the tropics, based primarily on the effects of warming on species distributional ranges [21]. At a large geographical scale, the findings are broadly similar to those based on the species biogeography approach, even though the present projections are based on completely different mechanisms arising from food web processes widely held to govern empirical patterns of size spectra in the open ocean and shelf seas [24,49].

An advantage here is the inclusion of fishing effects, enabling the relative effects of climate change and fishing to be explored within and across size-structured ecosystems. Changes in primary production [47] and temperature [50] affected growth rates and fish production, altering the responses of ecosystems to fishing. Either low primary production or cold-water ecosystems conferred higher susceptibility to fishing effects, due to slow relative growth rates. Cold-water ecosystems with higher seasonal fluctuations have been previously described as less likely to sustain heavy exploitation [51]. Also in keeping with empirical studies of fish populations [52–54] and theoretical size-spectrum models [44,55], fishing effects caused ecosystems to become more variable through time, due to reductions in size structure and shifts towards smaller size and higher growth rates. For the same reason, heavily fished ecosystem states were less resilient to climate change compared with unexploited ecosystem states [56].

For 1992–2001, our models generated catches and growth rates that were broadly realistic when compared with reported catches and growth rates, keeping in mind that the true community-wide fishing mortality rates within these ecosystems are not well known and the landed catch data may be subject to misreporting and bias [57]. The size-based models with relatively limited parameter demands provided surprisingly good estimates of current catch from some of the EEZ, further emphasizing the dominant role of body size in accounting for patterns of predatory interaction and production in marine ecosystems. The weakness of the size-based perspective is that it does not provide predictions of catches from individual species and account for their responses to fishing, but this has to be considered in the context that long-term predictions of individual species dynamics; even when complex population models are developed, they can be unreliable [58]. For example, although total fish production in an ecosystem may be maintained, there may be significant and unpredictable switches in the species contributing. Because recruitment is not modelled at a population level and was held constant to facilitate cross-comparison, the resilience of the community to fishing is not recruitment limited; it does not consider the negative feedback that can result from the removal of highly fecund, large mature spawning fish. For this reason, the specific ecosystem responses associated with a given value of fishing mortality should not be taken in absolute terms and are presented for comparative purposes.

The approach also ignores energy inputs from sources of primary production other than phytoplankton (such as macroalgae, seagrasses and mangroves). Although these make a relatively small contribution globally, contributing to around 5.5 per cent of total marine primary production [59], they are locally and regionally important contributors to inshore fish production. If the underlying rules determining the links between primary production and fish production do not change markedly with a changing climate, then our capacity to predict future changes in fish production is largely predicated on our capacity to predict future changes in the primary production and the physical environment. We assumed a universal relationship between temperature and the activation energy of metabolism to predict temperature-dependent changes in feeding rates, which may underestimate the effects of warming. More complex species and size-specific empirical relationships with temperature and activation energies for different processes such as attack rates, handling times have been described in this issue [10,11].

Bearing in mind the earlier-mentioned strengths and limitations, the model results are of potential use for global-scale social–ecological analyses such as country-level metrics of vulnerability to climate change [2]. They may also be useful for establishing levels of threat and uncertainty in specific regions, if combined with other model predictions. For example, in the Indo-Pacific ecosystem, the EEZ surrounding the country of Palau experienced the greatest loss of primary production, potential fisheries production and an increase in susceptibility to overfishing. This region is located within the Indo-Pacific Biodiversity Triangle and has the highest richness of corals, molluscs, crustaceans, finfishes and chondrichthyans in the world. It has some of the highest catches of chondrichthyans, mainly through unregulated fisheries [60]. Our results are likely to underestimate climate impacts in these regions where there will be impacts on other sources of production. Furthermore, we considered only one possible forcing scenario taken from one global climate model, using a time slice approach. To fully quantify projected future changes in fish production, it is also necessary to consider the uncertainties associated with the forcing data used, the temporal and spatial scales, as well as the mechanisms included. A formal model ensemble approach (including alternative physical–biogeochemical and ecological models) combined with detailed empirical ground-truthing and retrospective analyses will improve our capabilities to gauge where the most important uncertainties lie. There is a clear need for much greater understanding of the effects of climate change within regional seas, at more localized scales than considered here, as well the role of human responses to change. Advancing these integrated areas of research alongside improving our mechanistic understanding of complex ecological communities across spatial scales will help to elucidate sustainability of fisheries for the larger human population and warmer oceans of the future [3,45,61].
